# Sex differences in the long-term tolerability of BNT162b2 in children and adolescents

**DOI:** 10.1186/s40348-026-00243-2

**Published:** 2026-05-27

**Authors:** Jeanne Moor, Vivien Grieshaber, Nicole Toepfner, Sarah Holzwarth, Matthias B. Moor, Karolina Kublickiene, Christoph Strumann, Cho-Ming Chao

**Affiliations:** 1 Chair for Gender Medicine, University of Zurich, Zurich, Switzerland; 2 Institute of Primary Health Care (BIHAM), University of Bern, Bern, Switzerland; 3 CLINTEC Division of Renal Medicine, Karolinska Institutet, Stockholm, Sweden; 4 Faculty of Health, Witten/Herdecke University, Witten, Germany; 5 Department of Pediatric and Adolescent Surgery, Pediatric Urology and Pediatric Traumatology, Asklepios Children’s Hospital Sankt Augustin, Sankt Augustin, Germany; 6 Department of Pediatrics, Faculty of Medicine and University Hospital Carl Gustav Carus, Dresden University of Technology, Dresden, Germany; 7 Department of Nephrology and Hypertension, Inselspital University Hospital Bern, Bern, Switzerland; 8 University Medical Center Schleswig-Holstein, Campus Luebeck, Luebeck, Germany; 9 Cardio-Pulmonary Institute (CPI), Universities of Giessen and Marburg Lung Center (UGMLC), Member of the German Center for Lung Research (DZL), Justus Liebig University Giessen, Giessen, Germany; 10 Centre for Clinical and Translational Research (CCTR), Helios University Hospital Wuppertal, Witten/Herdecke University, Wuppertal, Germany; 11 St. Louise Children’s Hospital, Paderborn, Germany

**Keywords:** MRNA Vaccine, Sex differences, BNT162b2, Female, Vaccine reaction, Sex-specific

## Abstract

**Background:**

Sex differences exist not only in the efficacy but also in adverse event rates of many vaccines. Here we compared the sex differences in the tolerability of BNT162b2 in children younger than 18 years in Germany.

**Methods:**

A post-hoc analysis of retrospectively collected tolerability data, collected through an authentication-based survey of legal guardians of children vaccinated with BNT162b2 under the age of 18 years (CoVacU18-study). Primary outcome was the frequency of the four most common post-vaccination symptom categories (local, general, musculoskeletal symptoms, fever) reported after the 1st-4th doses. Data were analyzed according to sex in bivariate analyses and regression models adjusting for age, weight, and dosage. Interactions between sex and age group effects on post-vaccination symptoms were assessed. An active-comparator analysis was applied to compare post-vaccination symptoms after BNT162b2 versus non-SARS-CoV-2 vaccines.

**Results:**

Three thousand two hundred twenty-eight participants (median age 5.7 years, male 49.6%). In logistic regression, female sex was associated with higher odds of local symptoms (OR = 1.28 [95% CI: 1.17–1.40], *p* < 0.05), general symptoms (OR = 1.27 [1.13–1.44], *p* < 0.05), and musculoskeletal symptoms (OR = 1.27 [1.03–1.56], *p* < 0.05). Interactions between sex and age groups existed for post-vaccination local symptoms. Following non-BNT162b2 childhood vaccinations, female sex was not associated with odds of any post-vaccination symptoms. No relevant interaction existed between vaccine types (BNT162b2 vs non-BNT162b2) and sex for the association with post-vaccination symptoms.

**Conclusion:**

Sex differences exist in post-vaccination symptoms after BNT162b2 administration in young children and adolescents. These are of importance for the conception of approval studies, for post-vaccination monitoring and for future vaccination strategies.

**Supplementary Information:**

The online version contains supplementary material available at 10.1186/s40348-026-00243-2.

## Introduction

Human immunological reactions to self- and non-self-antigens are not only influenced by factors such as age, genetics and chronic diseases but by biological sex [1, 2]. Sex-specific differences exist in the frequency distribution of autoimmune and oncological diseases as well as in the immunological response to infections and vaccinations. For several vaccines, including the mRNA-based COVID-19 vaccine BNT162b2, it has been shown that the measured immune responses are more pronounced in females than in males [3–5]. Underlying mechanisms described include influences of the genes encoded on the gonosomes, steroid receptors as well as concentration of steroid hormones, whose age-related variability influences susceptibility to diseases [6–12]. Unfortunately, studies revealed sex differences in frequency of vaccine-related side effects. As with other vaccines, vaccine surveillance data and pooled analyses of cross-sectional studies have shown that females are more likely to experience any adverse events after vaccination with the Pfizer-BioNTech vaccine (Comirnaty®), whereas individual serious adverse events such as myocarditis affect males more frequently [13–18]. Experiencing vaccine-related side effects can has a decisive influence on individual vaccination decision [19]. Since vaccinations are one of the most important and effective preventive measures for protection against infectious diseases, research on sex specific vaccine tolerability is not only relevant for individuals but also in public health policy. Despite knowledge about the strong immune response and the higher rates of side effects in females, dose determination in clinical trials is carried out independently of the biological sex and its influence on the adaptive immune response. The characteristics of sex specific immunological differences in childhood have not been sufficiently analyzed. In a previous study, we were able to show that sex-specific differences in vaccine tolerability also exists in very young children (< 5 years of age) and even before puberty [20]. To gain a better understanding of the vaccination experience in childhood the following study evaluates the vaccine tolerability of BNT162b2 based on the four most common symptom categories in four different age groups from 0–17-year-olds, thus including a period before and during puberty.

## Methods

### Study design

The present study was a post-hoc analysis of the CoVacU18 study, a retrospective cohort study that assessed self-reported tolerability of the mRNA-based COVID-19 vaccine BNT162b2 in children aged 0–17 years [21].

### Participants

Participants of the CoVacU18 study were recruited in cooperation with 10 outpatient doctors’ practices nationwide and the German vaccination promotion program “Bildung Aber Sicher”, whose databases contained the e-mail addresses of the legal guardians of vaccinated children. Allocation of an individual authentication code to parents or legal guardians enabled eligible-only, one-time, and pseudonymized participation in the study. All children who received at least one dose of BNT162b2 since 01 October 2021 and before the age of 18 years were eligible for inclusion. Exclusion criteria were duplicate entries that had overlaps in the variables age, sex, height, weight and authentication code unless the duplicate entry was explained as twins or triplets; missing/invalid authentication code; vaccination exclusively with vaccines other than BNT162b2. Follow-up vaccinations after the 18th year of life were excluded from our analysis [21].

### Study procedure

All study data were collected using an online survey addressed to the legal guardians of vaccinated children. The original survey was available from May 25th to July 11th 2023 on RED Cap (Research Electronic Data Capture) platform. All eligible participants were recruited via the e-mail addresses by which they had previously registered a child aged less than 18 years old for a vaccination with BNT162b2. The invitation e-mails were sent out three times to the legal guardians, containing an individual access code to allow pseudonymized but authentication-based survey participation only. The study was conducted according to the principles of the Declaration of Helsinki and is reported following the Strengthening the Reporting of Observational Studies in Epidemiology (STROBE) guideline for observational studies. The study protocol was assessed and approved by the Ethics Committee of University of Witten/Herdecke, Germany (vote No. S-61/2023). The study was registered in the German Clinical Trials Register (ID: DRKS00031994) [21].

### Variables

The data collection included demographic data (age, sex, weight, height) as well as comorbidities, long-term medication, number, date and batch number of COVID-19 vaccinations received. Next, questions covered post-vaccination symptoms occurring after BNT162b2 in 11 symptom categories (injection-site symptoms; general reactions; fever; any symptoms of the musculoskeletal, cardiovascular, pulmonary, otolaryngological, gastrointestinal, neurological, psychological or dermatological system including lymph node reactions) with close-ended and open-ended follow-up questions as described in the initial CoVacU18 study [21]. The specific symptoms included in each symptom category can be found in Supplemental Table 1. If a child received a non-SARS-CoV-2 vaccination up to 3 months prior to BNT162b2, tolerability was assessed regarding the same symptom categories to compare vaccine tolerability in an internal comparative analysis [21].

### Outcomes

The primary outcome of this post-hoc analysis was the sex-specific frequency of symptoms within the four categories of local reactions (injection site symptoms e.g. redness, pain, swelling), general symptoms (e.g. fatigue, feeling of weakness, general feeling of illness), musculoskeletal symptoms (e.g. muscle pain) and fever that occurred after the first to fourth BNT162b2 vaccination, further stratified by age group (< 2 years, 2–4 years, 5–11 years, 12–17 years). As secondary analyses, we tested whether an interaction between sex and age group was present in association with the primary outcome. Next, we investigated symptoms occurring after non-SARS-CoV-2 vaccinations, and we analyzed symptoms post-BNT162b2 administration in comparison with those occurring after non-SARS-CoV-2 vaccine administration in the period between January 2021 and December 2022.

### Statistical analyses

The present post-hoc analysis was performed without prior power analysis. Softwares STATA v. 18 and MATLAB v. R2025a were used. In the bivariate analysis, categorical data were compared by Fisher’s exact test or Chi-Squared test. Continuous data were analyzed by the unpaired, 2-tailed t-test or Wilcoxon rank sum test as appropriate. In the bivariate analysis, all p-values were adjusted for multiple testing for the number of considered variables using Bonferroni method.

Odds ratios (OR) for the occurrence of symptoms of the four categories (local symptoms, general symptoms, fever, musculoskeletal symptoms) were calculated using logistic regression models. As predictors we selected sex, age group (< 2 years, 2–4 years, 4–11 years and 12–17 years), vaccine dose administered (1st to 4th), and interaction between sex and age group. Models with and without the interaction term were compared by Likelihood Ratio test. All logistic regression models were controlled for age, height and weight for children. Multicollinearity among the predictors was assessed using Variance Inflation Factors (VIF). To ensure the robustness of our estimates despite the expected biological correlation between age, weight, and height, sensitivity analyses were performed by comparing models with and without these covariates to monitor for potential coefficient instability. Model calibration and specification were evaluated using the Hosmer–Lemeshow goodness-of-fit test (with 10 groups) and the Link Test [22] to verify the functional form. Model discrimination was quantified using the Area Under the ROC Curve (AUC), and the proportion of explained variation was estimated using Nagelkerke’s pseudo-R^2^.

An internal comparison analysis was conducted in a subgroup of children who received BNT162b2 as well as vaccines against other pathogens (e.g. against influenza) to contrast frequencies of postvaccination symptoms, using logistic regression models as described above but with an additional predictor (“BNT162b2” vs. “Non-BNT162b2”).

Significance level was set at *p* < 0.05. To handle missing observations, listwise deletion was applied. As a sensitivity analysis, we performed multiple imputation using all complete explanatory variables to verify the robustness of our findings.

## Results

### Study sample

The study population of the CoVacU18-study consisted of 3228 children, including 1618 (50.1%) girls and 1601 (49.6%) boys. No gender was specified for 9 participants. These participants were excluded from the post-hoc analysis. Median age was 5.7 years (interquartile range [IQR]: 3.4–9.5). Among the children, 396 (12.3%) were reported to have comorbidities and 246 (7.6%) were taking long-term medications [21]. Overall and sex-stratified demographics data are shown in Table 1. Distributional analysis using Shapiro–Wilk and Shapiro-Francia tests indicated that age, height, and weight did not follow a normal distribution. Additionally, outliers were identified within the height and weight datasets. Consequently, these variables were compared using non-parametric methods. No statistically significant differences were observed between groups for any demographic parameters. Of note, a sex difference was found in the fraction of children with pre-existing comorbidities, with 166 (10.3%) of girls and 229 (14.3%) of boys (*p* = 0.002). Missing data for all considered variables are shown in Supplemental Table 2.

**Table 1:** Sample characteristics, n (%)

	All*	**Female**	**Male**	
	***n* = 3228 (100%)**	***n* = 1618 (50.1%)**	***n* = 1601 (49.6%)**	***p*-value**^a^
Age, median (IQR), y	5.7 (3.4–9.5)	5.8 (3.3–9.5)	5.7 (3.4–9.5)	> 0.999^b^
Height, median (IQR), cm	116 (98–140)	117 (98–140)	116 (99–140)	> 0.999^b^
Weight, median (IQR), kg	20 (15–32)	20 (14–32)	20 (15–32)	> 0.999^b^
Comorbidities (yes)	396 (12.3)	166 (10.3)	229 (14.3)	**0.002**^**c**^
Long-term medication (yes)	246 (7.6)	106 (6.6)	138 (8.6)	0.137^c^

^*^Including participants of the CoVacU18-study who did not provide gender information

^a^Adjusted for multiple testing by Bonferroni correction

^b^Wilcoxon rank-sum test following the identification of non-normal distribution and presence of outliers. *P*-values > 0.05 for all group comparisons

^c^Pearson’s chi-squared test. *P*-values in bold are statistically significant (*p* < 0.05)

### Sex-stratified post-vaccination symptoms occurring after BNT162b2 administration

**Fig. 1:**
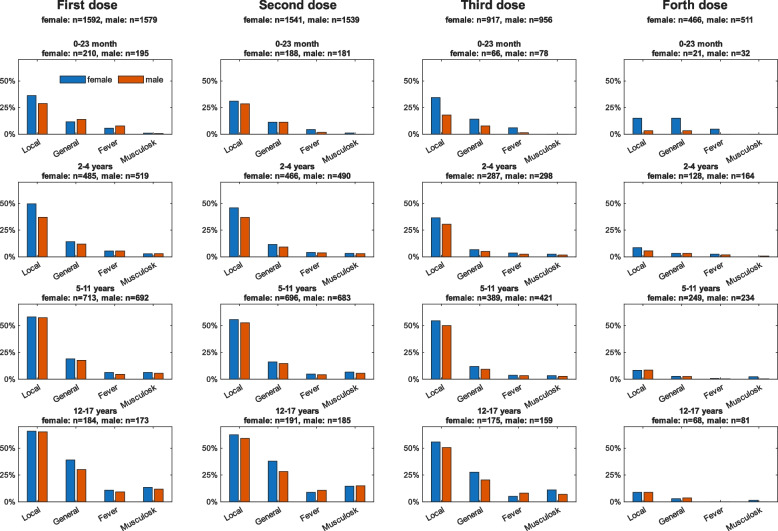
Frequency of the four most common post-vaccination symptoms reported after the 1st-4.^th^ dose of BNT162b2, stratified for age and sex, n (%)

We assessed the sex differences most common symptom categories reported in the original CoVacU18 study [21]. The proportions of children showing local symptoms, general symptoms, musculoskeletal symptoms and fever after the first dose of BNT162b2 administered are displayed in Fig. 1, with stratification for sex and age groups. Similarly, the frequencies of these four symptom categories reported after each of the first four doses of BNT162b2 administered are shown in Supplemental Table 3. There were sex differences in local injection-site symptoms for the first two BNT162b2 doses administered at the age group of 2–4 years, with 49.8% in girls vs. 37.2% in boys for the first dose (p < 0.001) and 45.8% in girls vs 36.9% in boys for the second dose of BNT162b2 (p = 0.021). No further gross sex differences were observed in the other symptoms categories.

In multivariable logistic regression with adjustment for four age categories, weight and height, we observed that after BNT162b2 vaccination, female sex was associated with reporting local symptoms (OR: 1.28 [95% CI: 1.17;1.40]), general symptoms (OR: 1.27 [95% CI: 1.13;1.44]) and also musculoskeletal symptoms (OR: 1.27 [95% CI: 1.03;1.56]) (Table 2). There was limited evidence of an association between female sex and fever (OR: 1.19 [95% CI: 0.97;1.45]) (Table 2). Further, for local symptoms an interaction between sex and age group existed with predominant reporting of local symptoms in females aged 2–4 years (Likelihood ratio test of difference in model upon including the interaction term: p < 0.05; Table 2). No interactions between sex and age groups reached significance for the other symptoms categories post BNT162b2 vaccination (Table 2). In a logistic regression approach stratified by age groups (Supplemental Table 4), we found that female sex was associated with a higher probability of local symptoms in children aged < 2 years (OR: 1.66 [95% CI: 1.24;2.22]) and in children aged 5–11 years (OR: 1.02 [95% CI: 1.01;1.03)]. Female sex was associated with fever in children aged 5–11 years (OR: 1.02 [95% CI: 1.00;1.03)]. Female sex was associated with a lower probability of post-BNT162b2 musculoskeletal symptoms in children aged 2–4 years (OR: 0.84 [95% CI: 0.74;0.96]), as shown in Supplemental Table 4. Our results remained highly robust when controlling for comorbidities and long-term medication, with chronic conditions only significantly affecting local symptoms and fever.

**Table 2:** Multivariable logistic regression of post-BNT162b2 symptoms, OR (95% CI)

n/N (%)	**local symptoms**		**general symptoms**		**fever**		**musculoskeletal symptoms**	
	3996/9102 (43.9)		1290/9069 (14.2)		432/9178 (4.7)		409/9041 (4.5)	
Model	Model 1	Model 2	Model 1	Model 2	Model 1	Model 2	Model 1	Model 2
Dosages (reference: First Dose)								
Second Dose	**0.88 (0.79—0.97)**	**0.88 (0.79—0.97)**	**0.83 (0.73—0.96)**	**0.83 (0.73—0.96)**	**0.76 (0.61—0.95)**	**0.76 (0.61—0.95)**	1.07 (0.85—1.35)	1.07 (0.85—1.35)
Third Dose	**0.73 (0.65—0.82)**	**0.73 (0.65—0.82)**	**0.55 (0.46—0.65)**	**0.54 (0.46—0.65)**	**0.56 (0.42—0.74)**	**0.56 (0.42—0.74)**	**0.62 (0.46—0.84)**	**0.62 (0.46—0.84)**
Fourth Dose	**0.06 (0.05—0.08)**	**0.06 (0.05—0.08)**	**0.12 (0.08—0.18)**	**0.12 (0.08—0.18)**	**0.11 (0.05—0.23)**	**0.10 (0.05—0.22)**	**0.16 (0.08—0.32)**	**0.16 (0.08—0.32)**
Height, median (IQR), cm	1.00 (0.99—1.01)	1.00 (0.99—1.01)	1.00 (0.99—1.01)	1.00 (0.99—1.01)	1.01 (0.99—1.02)	1.00 (0.99—1.02)	0.99 (0.98—1.01)	0.99 (0.98—1.01)
Weight, median (IQR), kg	**1.00 (1.00—1.01)**	**1.00 (1.00—1.01)**	**1.01 (1.01—1.02)**	**1.01 (1.01—1.02)**	0.99 (0.98—1.00)	0.99 (0.98—1.00)	**1.02 (1.01—1.03)**	**1.02 (1.01—1.03)**
female sex (reference: male)	**1.28 (1.17—1.40)**	1.14 (0.90—1.45)	**1.27 (1.13—1.44)**	**1.56 (1.20—2.03)**	1.19 (0.97—1.45)	0.83 (0.54—1.27)	**1.27 (1.03—1.56)**	1.23 (0.85—1.79)
female sex X age (< 2 years)		1.32 (0.91—1.92)		0.68 (0.42—1.09)		1.70 (0.81—3.60)		3.31 (0.36—30.7)
female sex X age (2–4 years)		**1.36 (1.02—1.81)**		0.80 (0.56—1.16)		1.38 (0.77—2.45)		0.85 (0.46—1.58)
female sex X age (5–11 years)		0.98 (0.74—1.28)		0.77 (0.56—1.06)		**1.71 (1.00—2.90)**		1.09 (0.68—1.76)
age groups (reference: 12–17 years)								
age (< 2 years)	**0.42 (0.29—0.60)**	**0.36 (0.24—0.55)**	0.73 (0.45—1.20)	0.91 (0.52—1.60)	**0.35 (0.16—0.78)**	**0.26 (0.11—0.64)**	**0.13 (0.04—0.40)**	**0.06 (0.01—0.46)**
age (2–4 years)	**0.60 (0.45—0.79)**	**0.51 (0.37—0.70)**	**0.48 (0.33—0.69)**	**0.54 (0.36—0.83)**	**0.35 (0.19—0.65)**	**0.29 (0.15—0.58)**	**0.43 (0.24—0.79)**	**0.47 (0.24—0.93)**
age (5–11 years)	0.95 (0.78—1.16)	0.96 (0.75—1.23)	**0.53 (0.42—0.68)**	**0.62 (0.46—0.84)**	**0.46 (0.30—0.68)**	**0.34 (0.21—0.56)**	**0.60 (0.42—0.86)**	**0.57 (0.36—0.90)**
intercept	0.77 (0.41—1.45)	0.81 (0.42—1.54)	**0.08 (0.03—0.19)**	**0.07 (0.03—0.17)**	0.25 (0.06—1.01)	0.31 (0.08—1.24)	**0.01 (0.00—0.05)**	**0.01 (0.00—0.05)**
Model diagnostics								
Nagelkerke R^2^ (in %)	15.3	15.4	9.1	9.2	3.8	3.9	9.4	9.5
Area under ROC curve	0.677	0.678	0.678	0.679	0.640	0.642	0.727	0.727
Hosmer–Lemeshow χ^2^ (pvalue)	12.9 (0.1163)	5.8 (0.6746)	3.9 (0.8648)	4.8 (0.7810)	4.6 (0.8011)	3.4 (0.909)	4.1 (0.846)	2.3 (0.9723)
LR-Test (pvalue)	11.96 (0.0075)		3.53 (0.3175)		4.27 (0.2342)		2.06 (0.5594)	
Observations	8848		8825		8917		8791	
children	3090		3077		3116		3063	
n/N (%)—sample	3879/8848 (43.8)		1252/8825 (14.2)		410/8917 (4.6)		392/8791 (4.5)	

Models 1 included no interaction terms. Models 2 included the interaction between sex and age group. Likelihood ratio tests (LR-Tests) determined if inclusion of the interaction term significantly improved the model fit

Bold entries are statistically significant (*p* < 0.05)

### Sex differences in symptoms reported after non-BNT162b2 vaccinations

Next, we aimed to determine if sex was associated with symptoms occurring after administration of Non-BNT162b2 vaccines directed against pathogens other than SARS -CoV-2 at any time point in the three months prior to a BNT162b2 vaccination. Overall, for 1244 (38.6%) children Non-BNT162b2 vaccinations were reported to be administered in the three months’ time frame. The most frequent vaccine antigens were influenza in 662 (20.6%) or combinations of tetanus, diphtheria, pertussis with/without poliovirus in 286 (8.9%) (Supplemental Table 5). However, using logistic regression models we found no evidence that fever, general, local or musculoskeletal symptoms depended on sex (Supplemental Table 7).

### Active-comparator analysis of symptoms occurring after BNT162b2 vs. Non-BNT162b2 vaccinations

Finally, we conducted an internal-comparator analysis of symptoms reported in a cohort of up to 2695 children who had received at least one BNT162b2 mRNA vaccine within the last 3 months with those occurring in 1244 individuals of the same population after receiving the non-BNT162b2 vaccinations listed in Supplemental Table 5. In a logistic regression model of all vaccinations combined and with adjustment for potential confounding of age, weight and height, female sex was associated with a higher probability of local symptoms (OR: 1.22 [95% CI: 1.06;1.40]), but there was not significant association of sex with general or musculoskeletal symptoms or fever (Table 3). In summary, these results indicate a modest sex effect favoring post-vaccination symptoms at the injection site in girls across the entire dataset of BNT162b2 and Non-BNT162b2 vaccinations.

**Table 3:** Comparison of symptoms occurring after BNT162b2 and after non-BNT162b2 vaccinations, OR (95% CI)

Total, n/N (%)	**local symptoms**		**general symptoms**		**fever**		**musculoskeletal symptoms**		
	1873/3771 (49.7)		804/3758 (21.4)		370/3795 (9.7)		196/3746 (5.2)		
BNT162b2, n/N (%)	1591/2751 (57.8)		625/2738 (22.8)		259/2775 (9.3)		196/2726 (7.2)		
non-BNT162b2, n/N (%)	282/1020 (27.6)		179/1020 (17.5)		111/1020 (10.9)		0/1020 (0)		
Model	Model 1	Model 2	Model 1	Model 2	Model 1	Model 2	Model 1	Model 2	
BNT162b2 vs. non-BNT162b2	**3.53 (3.00—4.15)**	**3.26 (2.58—4.12)**	**1.42 (1.17—1.72)**	1.30 (0.98—1.71)	0.96 (0.75—1.22)	0.78 (0.55—1.09)	-		-
female sex	**1.22 (1.06—1.40)**	1.09 (0.82—1.45)	1.13 (0.96—1.33)	0.99 (0.71—1.38)	1.02 (0.82—1.27)	0.76 (0.51—1.15)	1.11 (0.82—1.51)		1.11 (0.82—1.51)
female sex X BNT162b2		1.16 (0.84—1.60)		1.19 (0.81—1.74)		1.51 (0.93—2.46)			-
Height, median (IQR), cm	1.00 (0.99—1.01)	1.00 (0.99—1.01)	1.00 (0.98—1.01)	1.00 (0.98—1.01)	1.01 (0.99—1.03)	1.01 (0.99—1.03)	0.99 (0.97—1.01)		0.99 (0.97—1.01)
Weight, median (IQR), kg	1.00 (0.99—1.01)	1.00 (0.99—1.01)	1.01 (1.00—1.02)	1.01 (1.00—1.02)	0.99 (0.98—1.01)	0.99 (0.98—1.01)	1.01 (1.00—1.03)		1.01 (1.00—1.03)
age groups (reference: 12–17 years)									
age (< 2 years)	**0.50 (0.29—0.88)**	**0.50 (0.29—0.88)**	1.30 (0.68—2.47)	1.30 (0.68—2.47)	1.87 (0.75—4.70)	1.87 (0.75—4.70)	**0.07 (0.01—0.38)**		**0.07 (0.01—0.38)**
age (2–4 years)	**0.54 (0.35—0.84)**	**0.54 (0.35—0.84)**	**0.50 (0.30—0.83)**	**0.50 (0.30—0.82)**	0.75 (0.36—1.59)	0.75 (0.36—1.58)	**0.35 (0.15—0.84)**		**0.35 (0.15—0.84)**
age (5–11 years)	0.88 (0.64—1.20)	0.87 (0.64—1.20)	**0.55 (0.39—0.77)**	**0.55 (0.39—0.77)**	0.72 (0.43—1.20)	0.71 (0.43—1.20)	**0.49 (0.28—0.85)**		**0.49 (0.28—0.85)**
intercept	0.45 (0.17—1.21)	0.48 (0.17—1.29)	**0.09 (0.03—0.32)**	**0.10 (0.03—0.34)**	**0.17 (0.03—0.87)**	0.20 (0.04—1.01)	**0.04 (0.00—0.33)**		**0.04 (0.00—0.33)**
Model diagnostics									
Nagelkerke R2 (in %)	12.5	12.5	4.8	4.8	3.3	3.5	7.8		7.8
Area under ROC curve	0.677	0.677	0.619	0.619	0.608	0.619	0.683		0.683
Hosmer–Lemeshow χ2 (pvalue)	38.8 (< 0.001)	32.8 (< 0.001)	40.9 (< 0.001)	51.8 (< 0.001)	10.7 (0.2218)	4.71 (0.7878)	4.4 (0.8211)		4.4 (0.8211)
LR-Test (p-value)	0.79 (0.3735)		0.79 (0.3727)		2.77 (0.096)		-		
observations	3661		3651		3683		2648		
children	2672		2664		2694		2648		

Models 1 included no interaction terms. Models 2 included the interaction between sex and BNT162b2. Likelihood ratio tests (LR-Tests) determined if inclusion of the interaction term significantly improved the model fit

Bold entries are statistically significant (*p* < 0.05)

## Discussion

### Principal findings

The present study aimed to define the sex differences in the symptoms occurring post BNT162b2 vaccination across childhood in a retrospective authentication-based survey study. The present data reveal that female sex was associated with local injection-site symptoms, general and musculoskeletal symptoms occurring after BNT162b2 administration. It should be noted, however, that this is not statistically significant across all age groups. Further, in the present dataset the administration of other vaccines in the preceding three months were not associated with a post-vaccination sex-specific occurrence of symptoms in children.

### Interpretation of findings

The data of the present study reproduce findings on female-predominant injection-site symptoms of BNT162b2 reported by the CoVacU5 study [23].

The phenomenon of female-predominant injection-site symptoms is well established for many vaccine types over the years, observed in observational data and randomized studies as well as monitoring databases of adverse reactions to vaccines [24–26].

While the underlying mechanisms are not clear, they might include non-antigenic factors of vaccine formulation including adjuvant, or also a sex-specific nociception response to the physical trauma of the needle trauma. Several sex differences have been reported for nociception, but most sex-disaggregated vaccination data emerged only recently and leave substantial room for mechanistic investigations [27, 28].

### Strengths and limitations

Strengths of this study include that the sample consisted of children from all age groups, and the use of authentication codes for verified access of individuals truly registering their children for BNT162b2 vaccination. A potential limitation of our analysis is that children receiving non-BNT162b2 vaccines may systematically differ from those receiving BNT162b2. To address this, we compared sample characteristics and identified significant differences in age distribution, particularly with a higher proportion of children under 2 years and fewer aged 5–11 in the non-BNT162b2 group (see Table 6 in the supplements). However, we performed a robustness check by restricting the analysis to children who received both vaccine types, effectively using each subject as their own control. This analysis confirmed our primary findings and suggests that the observed variations in characteristics did not substantially bias the overall results.

Limitations include the smaller sample size overall in comparison to our previous CoVacU5 study [22]: Especially the data collected on non-BNT162b2 vaccinations were of limited power to detect sex differences, as inherent to the increasing age of children fewer childhood vaccines are administered per vaccination schedule. Nevertheless, the present data capture the key sex-disaggregated findings of previous CoVacU5 study [20, 23]. An alternative explanation, may be that the higher rate of reported post-vaccination side effects among girls may also be due to sociological factors, as the reports were provided by their legal guardians. However, this alternative interpretation should be weighed against the plethora of data demonstrating female-specific increased probability of injection-site reactions after vaccination [24, 25]. Furthermore, given that our study evaluates four distinct outcomes alongside several subgroup and interaction analyses, the potential for Type I error inflation cannot be entirely ruled out. Although these analyses were embedded within a unified multivariate regression framework to minimize false positives, the corresponding results should be interpreted with appropriate caution. Other limitations are that no causality can be established from the present observational data. Given the long interval between the vaccination date and the survey period, there is a risk of recall bias, which may be significantly influenced by the severity of symptoms, the child’s age, and the number of vaccine doses administered. Sensitivity analyses revealed no significant association between the reported rates of adverse effects and the timing of vaccination.

In addition, a wide variety of vaccines were included in the comparative analysis. No direct comparison analysis of the individual non-SARS-CoV-2 vaccines with BNT162b2 has been conducted. Therefore, the results of the comparative analysis must be interpreted with caution due to the heterogeneity of the vaccines in the non-SARS-CoV-2 group. For example, the meningococcal B vaccine exhibits comparatively higher reactogenicity than the acellular tetanus vaccine.

Participation in the study was voluntary, which – depending on the postvaccination symptoms occurred – could indicate either a high willingness among participants (a negative effect on tolerability) or support for vaccination (a positive effect on tolerability). From a methodological perspective, recruitment through vaccination sites offering off-label vaccination may lead to selection bias. Finally, although the participant recruiting for CoVacU18 was launched in a novel call almost 1 year after the CoVacU5 participants were recruited, a potential overlap of some participants between CoVacU5 and CoVacU18 cannot be excluded.

### Implications of findings

We here establish across all age groups of children that guardians of females are more likely to report local injection-site symptoms post-BNT162b2 vaccination compared to males. Transparent and clear communication of the female-predominant local injection reaction occurrence may increase the general public’s adherence and trust in vaccination programs, including administration of BNT162b2 in children. As suggested, the present data suggest that sex-specific vaccine formulation may be evaluated for future vaccine licensure studies as a large part of the population is vaccinated [20].

## Conclusion

Throughout childhood, BNT162b2 vaccination is associated with an increased probability of local injection-site symptoms in females compared to males. The present data call for a more systematic sex disaggregation of vaccination research data. Future studies should address the potential underlying mechanisms and may as a consequence consider the development and evaluation sex-adapted formulations of future vaccines.

## Supplementary Information

Supplementary Material 1.

## Data Availability

The datasets generated and analysed during the current study are not publicly available due to protection of participant’s anonymity.

## Abbreviations

OR: Odds ratio
95%-CI: 95-%-Confidence interval
