# Supplementary material for: Sex differences in the long-term tolerability of BNT162b2 in children and adolescents
Source: Mol Cell Pediatr. 2026 May 27;13:30. doi: 10.1186/s40348-026-00243-2 (PMC13212794; doi:10.1186/s40348-026-00243-2)
Supplement: Supplementary file 1 — Supplementary Material 1. [file 40348_2026_243_MOESM1_ESM.docx]

**Supplementary Online Content**

[**Supplemental Table 1.** Symptom categories and specific symptoms 2](#_Toc227659377)

[**Supplemental Table *2*.** Missing variables, n/N (%) 3](#_Toc227659378)

[**Supplemental Table *3.*** Post-vaccination symptoms after BNT162b2 vaccine, n/N (%) 4](#_Toc227659379)

[**Supplemental Table *4***. Multivariable logistic regression of post-BNT162b2 symptoms (age stratified), OR (95% CI) 5](#_Toc227659380)

[**Supplemental Table *5***. Non-BNT162b2 vaccines, n (%) 6](#_Toc227659381)

[**Supplemental Table *6***. Sample characteristics of children receiving BNT162b2 versus non-BNT162b2 vaccines 6](#_Toc227659382)

[**Supplemental Table *7***. Comparison of symptoms occurring after BNT162b2 and after non-BNT162b2 vaccinations (only children with non-BNT162b2 vaccinations), OR (95% CI) 7](#_Toc227659383)

| **Category** | **Symptoms** |
| --- | --- |
| Local | Redness, swelling, pain, others. |
| Fever | - |
| General | Chills, flashes, fatigue, flu-like symptoms, weakness, general feeling of illness, PIMS (pediatric inflammatory multisystem syndrome), others. |
| Musculoskeletal | Muscle weakness, muscle pain, muscle twitching, neck/back pain, pain in the arms, pain in the legs, joint pain, joint swelling, pain in the limbs, other |
| Gastrointestinal | Abdominal pain, nausea/vomiting, constipation, diarrhea, other changes in bowel habits, unintentional weight loss, unintentional weight gain, other |
| Ear-Nose-Throat | Nosebleeds, redness of the oral mucosa, swelling of the tongue, swelling of the lips, abnormal sensations in the mouth, toothache, bleeding gums, sore throat, earache, hoarseness, facial swelling, swollen lymph nodes, impaired sense of smell, impaired sense of taste, others |
| Pulmonary | Cough, irregular breathing, rapid breathing, exertional dyspnea, dyspnea at rest, other |
| Cardiovascular | Circulatory collapse/syncope, rapid heartbeat/palpitations, heart/chest pain, chest tightness, cold hands/feet, discoloration of the hands/feet, thrombosis/embolism, others |
| Neurological | Headache, dizziness, sensory disturbances, movement disorders, loss of consciousness, shooting pain, seizure, facial paralysis, others |
| Psychological | Difficulty concentrating, memory problems, sleep disturbances, aggressive behavior, nervousness, hyperactivity, anxious behavior, sadness/depression, significant mood swings, others |
| Dermatological | Localized rash, generalized rash, swollen lymph nodes, tender lymph nodes, widespread skin redness, other skin discoloration, hives, blisters, petechiae, bruises, dry patches of skin, eczema, sores, itching, other |

## **Supplemental Table 1.** Symptom categories and specific symptoms

## **Supplemental Table *2*.** Missing variables, n/N (%)

| **Variable** | **Missing, n (%)** |  |  |
| --- | --- | --- | --- |
|  | n=3228 |  |  |
| female sex | 9 (0.3) |  |  |
| Height | 78 (2.4) |  |  |
| Weight | 81 (2.5) |  |  |
| Comorbidities | 0 (0) |  |  |
| Long-term medication | 4 (0.1) |  |  |
| Vaccination specific | First Vaccination | Second Vaccination | Third Vaccination |
|  | n=3228 | n=3152 | n=2105 |
| Age | 25 (0.8) | 37 (1.2) | 25 (1.2) |
| local symptoms | 43 (1.3) | 44 (1.4) | 174 (8.3) |
| general symptoms | 59 (1.8) | 59 (1.9) | 180 (8.6) |
| fever | 12 (0.4) | 16 (0.5) | 158 (7.5) |
| musculoskeletal symptoms | 71 (2.2) | 70 (2.2) | 183 (8.7) |
| Vaccination specific | Fourth Vaccination | Fifth Vaccination | Sixth Vaccination |
|  | n=1089 | n=262 | n=48 |
| Age | 23 (2.1) | 2 (0.8) | 1 (2.1) |
| local symptoms | 80 (7.4) | 28 (10.7) | 9 (18.8) |
| general symptoms | 79 (7.3) | 28 (10.7) | 9 (18.8) |
| fever | 71 (6.5) | 28 (10.7) | 9 (18.8) |
| musculoskeletal symptoms | 81 (7.4) | 29 (11.1) | 9 (18.8) |

## **Supplemental Table *3.*** Post-vaccination symptoms after BNT162b2 vaccine, n/N (%)

|  |  | **First Vaccination** | | | **Second Vaccination** | | | **Third Vaccination** | | | **Fourth Vaccination** | | |
| --- | --- | --- | --- | --- | --- | --- | --- | --- | --- | --- | --- | --- | --- |
| Age | Symptoms | Female | Male | p-val^a^ | Female | Male | p-val^a^ | Female | Male | p-val^a^ | Female | Male | p-val^a^ |
| < 2 years | Local | 75/207 (36.2) | 56/195 (28.7) | 0.4327 | 58/187 (31.0) | 51/180 (28.3) | >0.999 | 23/65 (35.4) | 14/79 (17.7) | **0.0631** | 3/20 (15) | 1/32 (3.1) | >0.999^b^ |
|  | General | 24/207 (11.6) | 27/193 (14.0) | >0.999 | 21/187 (11.2) | 20/179 (11.2) | >0.999 | 9/65 (13.8) | 6/79 (7.6) | 0.8868 | 3/20 (15) | 1/32 (3.1) | >0.999^b^ |
|  | Fever | 12/210 (5.7) | 15/195 (7.7) | >0.999 | 8/188 (4.3) | 3/180 (1.7) | 0.8829^b^ | 5/67 (7.5) | 1/79 (1.3) | 0.3764^b^ | 1/21 (4.8) | 0/32 (0) | - |
|  | Musculosk | 2/207 (1.0) | 1/194 (0.5) | >0.999^b^ | 2/187 (1.1) | 0/180 (0) | - | 0/65 (0) | 0/79 (0) | - | 0/20 (0) | 0/32 (0) | - |
| 2-4 years | Local | 241/484 (49.8) | 191/514 (37.2) | **<0.001** | 212/463 (45.8) | 180/488 (36.9) | **0.0212** | 107/291 (36.8) | 90/300 (30) | 0.3237 | 11/131 (8.4) | 9/162 (5.6) | >0.999 |
|  | General | 69/480 (14.4) | 61/510 (12.0) | >0.999 | 53/459 (11.5) | 44/486 (9.1) | 0.8275 | 19/290 (6.6) | 15/300 (5) | >0.999 | 4/131 (3.1) | 5/162 (3.1) | >0.999^b^ |
|  | Fever | 26/486 (5.3) | 28/518 (5.4) | >0.999 | 19/467 (4.1) | 18/491 (3.7) | >0.999 | 11/291 (3.8) | 7/303 (2.3) | >0.999 | 3/131 (2.3) | 3/164 (1.8) | >0.999^b^ |
|  | Musculosk | 13/477 (2.7) | 15/508 (3.0) | >0.999 | 14/456 (3.1) | 14/484 (2.9) | >0.999 | 7/289 (2.4) | 5/300 (1.7) | >0.999 | 0/130 (0) | 1/162 (0.6) | - |
| 5-11 years | Local | 412/709 (58.1) | 394/688 (57.3) | >0.999 | 384/691 (55.6) | 359/680 (52.8) | >0.999 | 214/392 (54.6) | 213/425 (50.1) | 0.8034 | 21/248 (8.5) | 20/235 (8.5) | >0.999 |
|  | General | 136/706 (19.3) | 121/684 (17.7) | >0.999 | 112/688 (16.3) | 99/675 (14.7) | >0.999 | 48/391 (12.3) | 39/421 (9.3) | 0.6620 | 7/247 (2.8) | 6/236 (2.5) | >0.999 |
|  | Fever | 46/717 (6.4) | 32/694 (4.6) | 0.5523 | 34/697 (4.9) | 30/684 (4.4) | >0.999 | 17/394 (4.3) | 14/427 (3.3) | >0.999 | 2/250 (0.8) | 1/238 (0.4) | >0.999^b^ |
|  | Musculosk | 47/703 (6.7) | 39/685 (5.7) | >0.999 | 46/685 (6.7) | 38/677 (5.6) | >0.999 | 14/390 (3.6) | 12/424 (2.8) | >0.999 | 6/247 (2.4) | 1/236 (0.4) | 0.4918^b^ |
| 12-17 years | Local | 122/186 (65.6) | 111/170 (65.3) | >0.999 | 119/191 (62.3) | 109/184 (59.2) | >0.999 | 98/176 (55.7) | 80/162 (49.4) | 0.9863 | 6/68 (8.8) | 7/81 (8.6) | >0.999 |
|  | General | 75/187 (40.1) | 51/170 (30.0) | 0.1838 | 72/192 (37.5) | 53/184 (28.8) | 0.2943 | 49/176 (27.8) | 33/163 (20.2) | 0.4110 | 2/68 (2.9) | 3/82 (3.7) | >0.999^b^ |
|  | Fever | 20/189 (10.6) | 16/173 (9.2) | >0.999 | 17/193 (8.8) | 21/188 (11.2) | >0.999 | 9/177 (5.1) | 13/165 (7.9) | >0.999 | 0/68 (0) | 0/82 (0) | - |
|  | Musculosk | 25/182 (13.7) | 20/169 (11.8) | >0.999 | 27/187 (14.4) | 28/183 (15.3) | >0.999 | 19/173 (11) | 11/162 (6.8) | 0.7171 | 1/67 (1.5) | 0/82 (0) | >0.999^b^ |
| unknown | Local | 4/7 (57.1) | 8/16 (50.0) | >0.999^b^ | 8/17 (47.1) | 6/15 (40.0) | >0.999 | 5/13 (38.5) | 4/9 (44.4) | >0.999^b^ | 0/9 (0) | 1/11 (9.1) | - |
|  | General | 1/7 (14.3) | 2/16 (12.5) | >0.999^b^ | 3/17 (17.6) | 2/14 (14.3) | >0.999^b^ | 3/12 (25) | 3/8 (37.5) | >0.999^b^ | 0/9 (0) | 1/11 (9.1) | - |
|  | Fever | 0/8 (0) | 1/17 (5.9) | - | 1/19 (5.3) | 1/17 (5.9) | >0.999^b^ | 0/15 (0) | 0/9 (0) | - | 0/9 (0) | 0/11 (0) | - |
|  | Musculosk | 1/7 (14.3) | 0/16 (0) | - | 1/17 (5.9) | 2/14 (14.3) | >0.999^b^ | 1/12 (8.3) | 1/8 (12.5) | >0.999^b^ | 0/9 (0) | 0/11 (0) | - |

^a^ Chi-square test unless otherwise stated and adjusted for multiple testing by Bonferroni correction. ^b^Fisher’s exact test. P-values in bold are statistically significant (p<0.05).

## **Supplemental Table *4***. Multivariable logistic regression of post-BNT162b2 symptoms (age stratified), OR (95% CI)

|  | **local symptoms** | **general symptoms** | **fever** | **musculoskeletal symptoms** |
| --- | --- | --- | --- | --- |
| **age (<2 years)** |  |  |  |  |
| Dosages (reference: First Dose) |  |  |  |  |
| Second Dose | 0.87 (0.63 - 1.18) | 0.86 (0.55 - 1.33) | 0.39 (0.18 - 0.81) | 0.77 (0.13 - 4.66) |
| Third Dose | 0.80 (0.52 - 1.24) | 0.79 (0.42 - 1.45) | 0.58 (0.23 - 1.45) | - |
| Fourth Dose | 0.21 (0.07 - 0.59) | 0.55 (0.19 - 1.61) | 0.25 (0.03 - 1.88) | - |
| female sex | **1.66 (1.24 - 2.22)** | 1.05 (0.70 - 1.58) | 1.38 (0.74 - 2.59) | 4.32 (0.47 - 40.10) |
| weight | 1.11 (0.99 - 1.25) | 1.00 (0.85 - 1.17) | 0.98 (0.77 - 1.25) | 0.78 (0.39 - 1.58) |
| height | 1.01 (0.98 - 1.04) | 1.00 (0.96 - 1.04) | 0.98 (0.93 - 1.04) | 1.13 (0.95 - 1.35) |
| Observations | 947 | 944 | 951 | 752 |
| children | 399 | 397 | 401 | 397 |
| **age (2-4 years)** |  |  |  |  |
| Dosages (reference: First Dose) | 0.92 (0.77 - 1.11) | 0.77 (0.58 - 1.03) | 0.69 (0.45 - 1.08) | 1.06 (0.61 - 1.85) |
| Second Dose | 0.70 (0.56 - 0.87) | 0.43 (0.29 - 0.64) | 0.44 (0.24 - 0.79) | 0.84 (0.42 - 1.70) |
| Third Dose | 0.10 (0.06 - 0.17) | 0.17 (0.08 - 0.38) | 0.27 (0.11 - 0.69) | 0.15 (0.02 - 1.10) |
| Fourth Dose | 1.57 (1.34 - 1.85) | 1.23 (0.95 - 1.60) | 1.12 (0.76 - 1.66) | 1.02 (0.62 - 1.68) |
| female sex | 0.96 (0.92 - 1.00) | 0.96 (0.89 - 1.02) | 1.03 (0.93 - 1.14) | **0.84 (0.74 - 0.96)** |
| weight | 1.03 (1.01 - 1.04) | 1.02 (0.99 - 1.04) | 0.97 (0.94 - 1.00) | 1.07 (1.03 - 1.11) |
| height | 0.92 (0.77 - 1.11) | 0.77 (0.58 - 1.03) | 0.69 (0.45 - 1.08) | 1.06 (0.61 - 1.85) |
| Observations | 2767 | 2755 | 2783 | 2743 |
| children | 1097 | 1089 | 1104 | 1085 |
| **age (5-11 years)** |  |  |  |  |
| Dosages (reference: First Dose) | 0.87 (0.75 - 1.01) | 0.82 (0.67 - 1.01) | 0.84 (0.60 - 1.19) | 1.02 (0.74 - 1.40) |
| Second Dose | 0.83 (0.70 - 0.99) | 0.57 (0.44 - 0.75) | 0.66 (0.42 - 1.03) | 0.54 (0.34 - 0.86) |
| Third Dose | 0.05 (0.04 - 0.08) | 0.12 (0.06 - 0.22) | 0.04 (0.01 - 0.28) | 0.26 (0.12 - 0.56) |
| Fourth Dose | 1.11 (0.97 - 1.27) | 1.21 (1.01 - 1.46) | 1.43 (1.04 - 1.96) | 1.34 (1.00 - 1.80) |
| female sex | **1.02 (1.01 - 1.03)** | 1.00 (0.98 - 1.02) | **1.02 (1.00 - 1.03)** | 1.00 (0.97 - 1.03) |
| weight | 0.99 (0.98 - 1.00) | 1.02 (1.01 - 1.03) | 1.00 (0.99 - 1.01) | 1.01 (1.00 - 1.03) |
| height | 0.87 (0.75 - 1.01) | 0.82 (0.67 - 1.01) | 0.84 (0.60 - 1.19) | 1.02 (0.74 - 1.40) |
| Observations | 3924 | 3909 | 3955 | 3902 |
| children | 1534 | 1527 | 1548 | 1522 |
| **age (12-17 years)** |  |  |  |  |
| Dosages (reference: First Dose) | 0.81 (0.59 - 1.09) | 0.91 (0.66 - 1.23) | 0.95 (0.59 - 1.55) | 1.21 (0.79 - 1.86) |
| Second Dose | 0.57 (0.42 - 0.78) | 0.56 (0.40 - 0.78) | 0.57 (0.32 - 0.99) | 0.68 (0.41 - 1.11) |
| Third Dose | 0.04 (0.02 - 0.08) | 0.06 (0.03 - 0.16) | - | 0.05 (0.01 - 0.36) |
| Fourth Dose | 1.06 (0.83 - 1.36) | 1.46 (1.12 - 1.90) | 0.78 (0.50 - 1.19) | 1.20 (0.82 - 1.75) |
| female sex | 1.00 (0.98 - 1.01) | 1.00 (0.98 - 1.01) | 0.98 (0.96 - 1.01) | 1.00 (0.98 - 1.02) |
| weight | 0.99 (0.98 - 1.01) | 1.00 (0.98 - 1.02) | 1.00 (0.97 - 1.03) | 1.02 (0.99 - 1.04) |
| height | 0.81 (0.59 - 1.09) | 0.91 (0.66 - 1.23) | 0.95 (0.59 - 1.55) | 1.21 (0.79 - 1.86) |
| Observations | 1191 | 1197 | 1061 | 1180 |
| children | 440 | 442 | 421 | 436 |

Bold entries are statistically significant (p<0.05)

## **Supplemental Table *5***. Non-BNT162b2 vaccines, n (%)

|  | **All** | **Female** | **Male** |  |
| --- | --- | --- | --- | --- |
|  | n=3219  (100%) | n=1618 (50.3%) | n=1601 (49.7%) | p-val^a^ |
| Non-BNT162b2 vaccines (yes) | 1244 (38.6) | 655 (40.5) | 589 (36.8) | **0.0315** |
| Influenza | 662 (20.6) | 352 (21.8) | 310 (19.4) | 0.6519 |
| Meningococcal | 214 (6.6) | 112 (6.9) | 102 (6.4) | >0.999 |
| Measles/mumps/rubella with/without chickenpox | 183 (5.7) | 99 (6.1) | 84 (5.2) | >0.999 |
| Tetanus/diphtheria/pertussis and/or pediatric polio | 286 (8.9) | 147 (9.1) | 139 (8.7) | >0.999 |
| Hepatitis A/B | 95 (3.0) | 49 (3.0) | 46 (2.9) | >0.999 |
| Human papillomavirus | 115 (3.6) | 70 (4.3) | 45 (2.8) | 0.1437 |
| Other | 320 (9.9) | 170 (10.5) | 150 (9.4) | >0.999 |

^a^Adjusted for multiple testing by Bonferroni correction. Bold entries are statistically significant (p<0.05)

## **Supplemental Table *6***. Sample characteristics of children receiving BNT162b2 versus non-BNT162b2 vaccines

|  | BNT162b2 | non-BNT162b2 | pvalue |
| --- | --- | --- | --- |
|  | n=2775 | n=1116 |  |
| female | 1390 (50.1) | 579 (51.9) | 0.312 |
| age groups |  |  |  |
| age (<2 years) | 316 (11.4) | 235 (21.1) | **<0.0001** |
| age (2-4 years) | 869 (31.3) | 337 (30.2) | 0.4952 |
| age (5-11 years) | 1251 (45.1) | 428 (38.4) | **0.0001** |
| age (12-17 years) | 339 (12.2) | 116 (10.4) | 0.1097 |
| Height, median, cm | 118.0 | 110.0 | 0.7887 |
| Weight, median, kg | 20.0 | 18.0 | 0.7205 |
| Comorbidities (yes) | 342 (12.3) | 163 (14.6) | 0.0555 |
| Long-term medication (yes) | 220 (7.9) | 100 (9) | 0.2868 |

Bold entries are statistically significant (p<0.05)

## **Supplemental Table *7***. Comparison of symptoms occurring after BNT162b2 and after non-BNT162b2 vaccinations (only children with non-BNT162b2 vaccinations), OR (95% CI)

|  | **local symptoms** | | **general symptoms** | | **fever** | | **musculoskeletal symptoms** | | |
| --- | --- | --- | --- | --- | --- | --- | --- | --- | --- |
| Total, n/N (%) | 877/2040 (43) | | 411/2037 (20.2) | | 211/2040 (10.3) | | 57/2037 (2.8) | | |
| BNT162b2, n/N (%) | 595/1020 (58.3) | | 232/1020 (22.8) | | 100/1020 (9.8) | | 57/1020 (5.6) | | |
| non-BNT162b2, n/N (%) | 282/1020 (27.6) | | 179/1020 (17.5) | | 111/1020 (10.9) | | 0/1020 (0) | | |
| Model | Model 1 | Model 2 | Model 1 | Model 2 | Model 1 | Model 2 | Model 1 | Model 2 | |
|  |  |  |  |  |  |  |  | |  |
| BNT162b2 vs. non-BNT162b2 | **3.77 (3.11 - 4.56)** | **3.73 (2.83 - 4.91)** | **1.39 (1.11 - 1.74)** | 1.25 (0.91 - 1.74) | 0.87 (0.65 - 1.17) | 0.72 (0.47 - 1.10) | - | | - |
| female sex | 1.09 (0.90 - 1.32) | 1.08 (0.81 - 1.43) | 1.11 (0.89 - 1.39) | 1.00 (0.72 - 1.40) | 0.92 (0.68 - 1.24) | 0.77 (0.51 - 1.16) | 1.23 (0.70 - 2.16) | | 1.23 (0.70 - 2.16) |
| female sex X BNT162b2 |  | 1.02 (0.70 - 1.50) |  | 1.21 (0.77 - 1.89) |  | 1.45 (0.80 - 2.64) |  | | - |
| Height, median (IQR), cm | 1.00 (0.98 - 1.02) | 1.00 (0.98 - 1.02) | 1.00 (0.98 - 1.03) | 1.00 (0.98 - 1.03) | 1.02 (0.98 - 1.05) | 1.02 (0.98 - 1.05) | 0.99 (0.94 - 1.04) | | 0.99 (0.94 - 1.04) |
| Weight, median (IQR), kg | 1.00 (0.98 - 1.01) | 1.00 (0.98 - 1.01) | 1.00 (0.99 - 1.02) | 1.00 (0.99 - 1.02) | 0.99 (0.97 - 1.01) | 0.99 (0.97 - 1.01) | 1.00 (0.97 - 1.04) | | 1.00 (0.97 - 1.04) |
| age groups (reference: 12-17 years) |  |  |  |  |  |  |  | |  |
| age (<2 years) | **0.3 (0.13 - 0.66)** | **0.3 (0.13 - 0.66)** | 1.43 (0.56 - 3.66) | 1.43 (0.56 - 3.66) | **4.6 (1.15 - 18.46)** | **4.61 (1.15 - 18.49)** | **0.04 (0.00 - 0.45)** | | **0.04 (0.00 - 0.45)** |
| age (2-4 years) | **0.33 (0.17 - 0.62)** | **0.33 (0.17 - 0.62)** | 0.67 (0.32 - 1.44) | 0.67 (0.32 - 1.44) | 1.58 (0.48 - 5.14) | 1.58 (0.48 - 5.14) | **0.17 (0.03 - 0.91)** | | **0.17 (0.03 - 0.91)** |
| age (5-11 years) | **0.62 (0.39 - 0.98)** | **0.62 (0.39 - 0.98)** | 0.75 (0.44 - 1.27) | 0.75 (0.44 - 1.27) | 1.58 (0.65 - 3.86) | 1.59 (0.65 - 3.86) | 0.36 (0.13 - 1.03) | | 0.36 (0.13 - 1.03) |
| intercept | 1.1 (0.25 - 4.89) | 1.11 (0.25 - 4.93) | 0.19 (0.03 - 1.11) | 0.2 (0.03 - 1.18) | 0.11 (0.01 - 1.27) | 0.12 (0.01 - 1.39) | 0.2 (0.00 - 12.06) | | 0.2 (0.00 - 12.06) |
| Model diagnostics |  |  |  |  |  |  |  | |  |
| Nagelkerke R2 (in %) | 15.1 | 15.1 | 2.8 | 2.9 | 6.6 | 6.8 | 8.7 | | 8.7 |
| Area under ROC curve | 0.695 | 0.695 | 0.606 | 0.609 | 0.649 | 0.655 | 0.700 | | 0.700 |
| Hosmer-Lemeshow χ2 (pvalue) | 14.2 (0.0761) | 14.1 (0.0790) | **32.7 (<0.001)** | **29.3 (<0.001)** | 11.2 (0.1934) | 2.6 (0.9570) | 4.0 (0.8604) | | 4.0 (0.8604) |
| LR-Test (pvalue) | 0.01 (0.9131) | | 0.7 (0.4026) | | 1.49 (0.2217) | | - | | |
| observations | 1978 | | 1976 | | 1978 | | 986 | | |
| children | 989 | | 988 | | 989 | | 986 | | |

Models 1 included no interaction terms. Models 2 included the interaction between sex and BNT162b2. Likelihood ratio tests (LR-Tests) determined if inclusion of the interaction term significantly improved the model fit. Bold entries are statistically significant (p<0.05)
